# Investigation of the Spatial Generation of Stimulated Raman Scattering Using Computer Simulation and Experimentation

**DOI:** 10.1177/00037028221123593

**Published:** 2022-10-24

**Authors:** Ronja Eriksson, Per Gren, Mikael Sjödahl, Kerstin Ramser

**Affiliations:** 1 Department of Engineering Science and Mathematics, 407846 Luleå University of Technology, Luleå, Sweden

**Keywords:** Stimulated Raman scattering, simulations, experiments, spatial distribution, spatial rate

## Abstract

Stimulated Raman scattering is a phenomenon with potential use in providing real-time molecular information in three-dimensions (3D) of a sample using imaging. For precise imaging, the knowledge about the spatial generation of stimulated Raman scattering is essential. To investigate the spatial behavior in an idealized case, computer simulations and experiments were performed. For the computer simulations, diffraction theory was used for the beam propagation complemented with nonlinear phase modulation describing the interaction between the light and matter. For the experiments, a volume of ethanol was illuminated by an expanded light beam and a plane inside the volume was imaged in transmission. For generating stimulated Raman scattering, a pump beam was focused into this volume and led to a beam dump after passing the volume. The pulse duration of the two beams were 6 ns and the pump beam energy ranged from 1 to 27 mJ. The effect of increasing pump power on the spatial distribution of the Raman gain and the spatial growth of the signal at different interaction lengths between the beam and the sample was investigated. The spatial width of the region where the stimulated Raman scattering signal was generated for experiments and simulation was 0.21 and 0.09 mm, respectively. The experimental and simulation results showed that most of the stimulated Raman scattering is generated close to the pump beam focus and the maximum peak of the Stokes intensity spatially comes shortly after the peak of the pump intensity.

## Graphical abstract

**Graphical Abstract:**
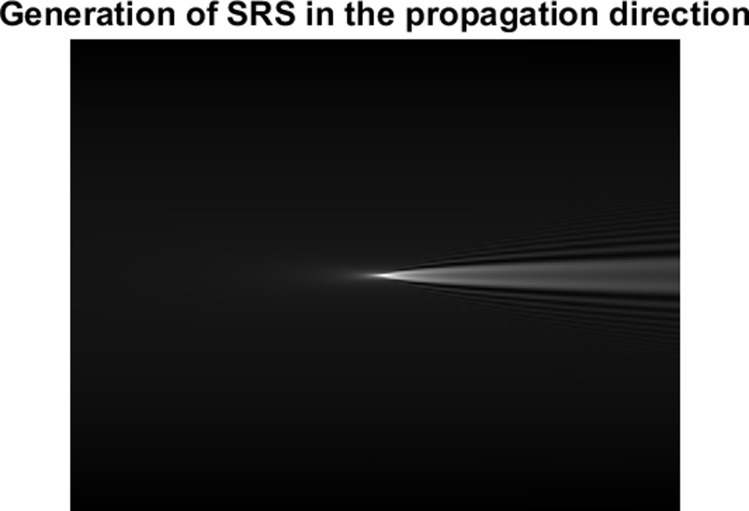

## Introduction

In industry and several areas of research such as biochemistry, medicine, and material science, there is a growing need for images that provide information about the three-dimensional (3D) structure, content as well as arrangement of molecular species of a sample. Fluorescence imaging^1–3^ can be used for in-vivo 3D imaging^4^ of living tissue and biological processes, but for the imaging to work the sample must often be stained with fluorochromes that can be toxic.^5^ Furthermore, fluorescence will alter the intrinsic properties of the sample and fluorescent bleaching is a common problem. Optical coherence tomography (OCT)^6–8^ is another imaging technique that is noninvasive and provides 3D information with high resolution. OCT relies on differences in scattering properties between the components of a sample and computational methods are applied to image the 3D structure of different materials and tissues. However, if two materials have almost the same scattering properties they cannot be distinguished, and this method does not provide molecular information.

To overcome those shortcomings, the nonlinear scattering phenomenon, stimulated Raman scattering (SRS),^9^ has during the last decade become a popular imaging technique since it is both fast and does not require labeling of the species of interest. SRS is generated by illuminating a sample with two laser beams, a pump beam with a frequency ν_P_ and a probe beam, usually called the Stokes beam, with the frequency ν_S_. The frequencies of the two beams are selected such that Δν = ν_P_ − ν_S_ corresponds to a Raman shift of the sample, causing a stimulated Raman gain (SRG) in the Stokes beam while the pump beam experiences a stimulated Raman loss (SRL). SRS microscopy is a commonly used imaging technique where the SRS image is constructed by point scanning the sample using a confocal microscope.^10^ This type of SRS microscopy has been used for live cell imaging,^11–13^ imaging of cancerous tissue,^14–16^ and volumetric 3D imaging^17–20^. Modifications can be made to the experimental setup to allow for probing of several Raman shifts simultaneously, this is usually called multiplex SRS (MSRS).^21–24^ There are examples where SRS imaging was applied without point scanning the sample. Amer et al.^25^ demonstrated a method where the polarization sensitivity of SRS was combined with polarization-resolved pulsed digital holography to record the SRS signal in a single shot hologram for time resolved imaging methane gas.

In our group, we are developing a new imaging technique that will fuse depth-gated interferometric imaging with SRS to provide 3D information of a sample without the need to point scan the sample. The depth resolution of interferometric imaging without SRS is dependent on depth gate set.^26^ However, if SRS is introduced, the resolution will depend on the size of the region where SRS is generated, which leads to the question where SRS is spatially generated if the pump light is focused into a volume illuminated with Stokes light. Carman et al.^27^ theoretically described the spatial shape of the Stokes light in the SRS generation process. A conclusion they reached was that the intensity maximum of the Stokes pulse spatially always comes after the intensity maximum of the pump pulse. Raymer and Mostowski^28^ theoretically described the buildup of SRS from the spontaneous Raman scattering initiated in the sample by the pump beam by considering spatial propagation in 1D. As a continuation, Raymer et al.^29^ described theoretically the coherence properties and spatial propagation of SRS in 3D. Lewis and Knudtson^30^ compared an analytic solution with experiments regarding the spatial growth rate of SRS. Lewis and Knudtson^31^ also measured the growth rate and the conversion efficiency of SRS for different liquids and complemented the previous analytic solution by taking the self-focusing due to the change in refractive index into consideration. In all these cases the generation of SRS is described in the form of Raman amplification.

This paper aims to investigate the spatial generation of SRS from the interaction between two beams propagating in a transparent medium by comparing computer simulations with experiments. The experimental setup and instrumentation are presented followed by the measurement principle. The theory and implementations of the computer simulations are then presented and are followed by results and discussion, and finally by the conclusions.

## Methods and Methods

### Instrumentation

**Figure 1.**
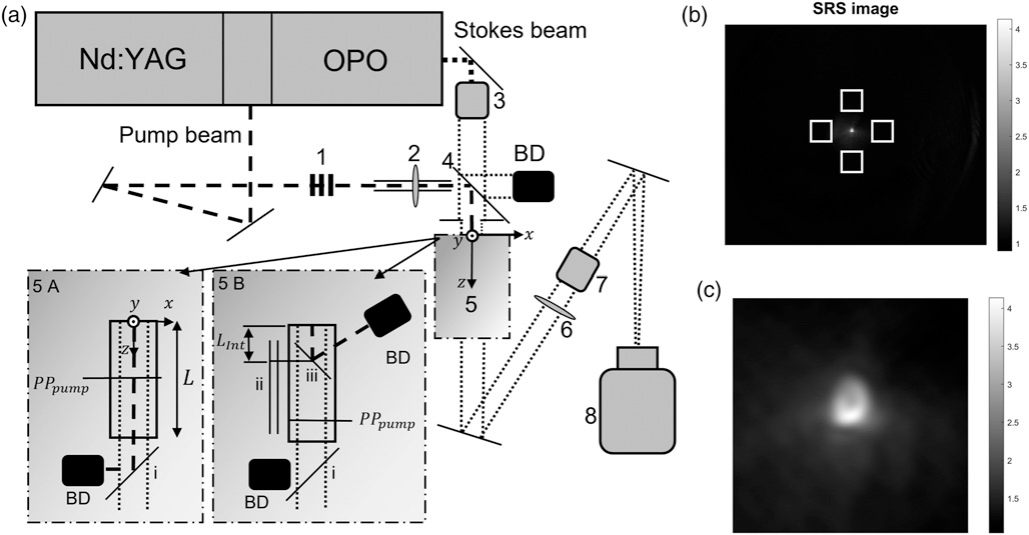
(a) Schematic of the experimental setup. The pump beam is represented by dashed lines and the Stokes beam by the dotted lines, respectively. The 532 nm pump beam passed through (1) a λ/2 plate and two thin film polarizers, (2) a focusing lens, (4) a dichroic mirror, and (5) the ethanol sample. The pump beam radius entering the sample was 2–2.5 mm. The 630.45 nm Stokes beam passed through (3) a 16× magnification telescope, (4) a dichroic mirror, (5) the sample, (6) a 250 mm imaging lens, (7) two edge filters removing 532 nm, before reaching, (8) a PCO edge camera with ND2.0, RG 630 nm, and BG39 filters. The Stokes beam radius entering the sample was 5 mm. Two sample setups were used. Panel 5A setup: The pump beam focus was positioned at *PP*_*pump*_, 85 mm from the entrance. After the cuvette, the pump beam was reflected into a beam dump (BD) using (i) a 532 nm reflective mirror. Panel 5B setup: The pump beam focus was positioned at *PP*_*pump*_, 123 mm from the sample entrance. (i) A 532 nm reflective mirror, (ii) a rail, and (iii) a dichroic 532 nm mirror that limited the interaction length *L*_*Int*_ between the pump and Stokes beams. The remaining pump light after the sample was reflected to a beam dump (BD) using a (ii) 532 nm reflective mirror. (b) An example of an SRS image, the areas used to calculate *M*_*back*_ of the Stokes light are marked by the white squares. The SRS amplification can be seen as a bright dot in the center. (c) A zoomed in image of the SRS. The two color bars in (b) and (c) show the ratio *I*_*sig*_/*I*_*ref*_.

The experimental setup is seen in Fig. 1a. The sample medium was 99.7% spectroscopic ethanol (Solveco AB, Sweden) placed in an in-house-made glass cuvette (35 × 70 × 170 mm^3^). The Raman line at 2934 cm^−1^ assigned to the CH_2_ asymmetric stretching of ethanol^32^ was targeted. The laser system was a Continuum PL 8000 system. A Q-switched 1064 nm neodymium-doped yttrium aluminum garnet (Nd:YAG) laser (Continuum Powerlite DLS) with a repetition rate of 10 Hz was first frequency doubled to 532 nm and then frequency tripled to 355 nm. The residual 532 nm vertically polarized beam remaining after the frequency tripling was used as the pump beam. The Stokes beam was produced by guiding the 355 nm beam into an optical parametric oscillator (Continuum Sunlite EX OPO) and tuned to 630.45 nm so that Δν corresponded to the 2934 cm^−1^ Raman shift. The pulse length of both the pump and Stokes beams was ∼6 ns.

The pump light was guided through a λ/2 plate and two thin film polarizers (1) to enable a seamless control of the pump pulse energy. The pump beam was then guided to a lens with focal length 250 mm mounted on a rail (2) that focused the beam into the sample (5). The radius of the pump light entering the sample was about 2–2.5 mm. The effective numerical aperture was about 0.03–0.04. The Stokes light was expanded 16 times (3) from its original size and combined with the pump light using (4) a dichroic mirror before entering the sample. An aperture was used to limit the radius of the Stokes beam entering the sample to about 5 mm, which caused the Stokes beam to have a top hat beam profile when hitting the camera. Note that in this way, the Stokes light covered a cylindrical volume of the sample while the pump light was focused to a spot within the illuminated volume. Two experimental setups were used. For experimental setup 1 (panel 5A in Fig. 1), the focal plane of the pump light, *PP*_pump_, was positioned in the middle of the cuvette, 85 mm from the entrance. The pump beam was separated from the Stokes beam after the sample using a reflective mirror (i). For experimental setup 2 (panel 5B in Fig. 1), *PP*_pump_ was located a distance 123 mm from the entrance and a dichroic 532 nm mirror mounted on a rail (ii) was submerged into the ethanol. This allowed for the removal of the pump beam from the sample, thus interrupting the SRS generation after a specific interaction length, L_*Int*_. Behind the sample, a 532 nm reflective mirror (i) was used to remove any remaining pump light from the Stokes beam. After passing through the sample the Stokes beam was guided to an imaging lens with focal length 250 mm (6) followed by two edge filters (7) before reaching a 16-bit pco.edge 5.5 camera (8). The camera is water-cooled, and the sensor has a size of 16.6 × 14 mm^2^, consisting of 2560 × 2160 pixels of pixel size 6.5 × 6.5 µm^2^. A neutral density filter (ND2.0) was used to prevent saturation of the detector. A BG39 filter was used to block infrared light that might come from the laser flashlamps, and an RG 630 nm filter was used to block wavelengths below 630 nm. The field of view in the setups, panels 5A and 5B, were around 9.8 × 8.3 mm^2^ with a pixel pitch around 3.7 μm.

### Measurement Principle

For all measurements performed, the plane *PP*_pump_ was imaged onto the camera using lens (6) in Fig. 1. The image magnification was kept constant between the two sample setups by adjusting the camera position. To create an SRS intensity image, *I*_SRS_, two camera images were used, an intensity signal image *I*_sig_ containing the Stokes light with the corresponding SRG and an intensity reference image *I*_*ref*_ containing the Stokes light without the SRG. Taking the ratio of the intensity, pixel by pixel, between the two images gave the SRS image, that is, *I*_SRS_ = *I*_sig_/*I*_*ref*_. Note that in an SRS image the background will have a value of 1 meanwhile the SRS signal will have a value larger than 1. *I*_SRS_ was calculated and analyzed using MATLAB tm, Mathwork. Before the calculation, *I*_sig_ and *I*_*ref*_ were filtered using a 2D Gaussian kernel with standard deviation σ, here the parameter was set to σ = 4 pixels. The exposure time of the camera was set to capture single pulses. However, the laser pulse energy varied from pulse to pulse and to average out some of these fluctuations the camera was set to produce an average image of 64 light pulses.

The maximum value of the SRS gain in an image, *G*_SRS_, was calculated by extracting the maximum pixel value, subtracting the mean value of the background, *M*_*back*_ and then multiplying the difference with 100, that is, *G*_SRS_ =(max(*I*_SRS_) – *M*_*back*_)100. Thus, if max(*I*_SRS_) = 2 and *M*_*back*_ = 1, the maximum gain would be *G*_SRS_ = (2–1)100 = 100%. Since the background was not completely even *M*_*back*_ was calculated by taking the mean of four areas of the background around the SRS signal. In Fig. 1b, an example of an SRS image can be seen. The areas used for *M*_*back*_ are marked as white squares. The bright area in the middle of the images is the SRG of the Stokes light. The outline of the background Stokes light can be seen as a large and weak circle. Fig. 1c shows a zoom-in copy of the amplified Stokes light. Value two of the color bar represents a gain of 100%.

### Theory and Approach to Simulations

**Figure 2.**
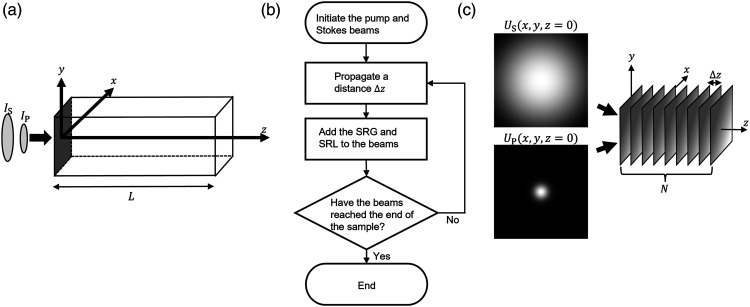
(a) A sketch of the sample with the coordinate system. The pump and Stokes beams, *I*_*P*_ and *I*_*S*_, respectively, enters the sample in the *x*,*y*-plane at *z* = 0. *L* is the sample length. The radius of *I*_*S*_ larger than the radius of *I*_*P*_. (b) A flowchart of the simulation process. (c) Numerical setup of the sample volume in (a) for the simulations. The volume is sliced into *N*, *x*,*y*-planes separated by a distance Δz. The initial Stokes beam can be seen to the upper left and the initial pump beam to the lower left.

To verify the positions of the SRS gain, the scenarios of the experimental setups in panels A and B in Fig. 1a were simulated. A sketch of the sample and coordinate system together with the direction of travel for the beams can be seen in Fig. 2a. In the simulation geometry, the pump and Stokes beams, with intensities *I*_P_ and *I*_S_, respectively, entered the sample at the plane *z* = 0. The beams then propagated in the *z*-direction through the sample and the nonlinear interaction between the medium and the beams generated an SRG in the Stokes light and an SRL in the pump light. The propagation and interaction of the beams were modeled using diffraction theory and phase modulation (PM) due to an induced Kerr effect, respectively. The two theories are described in more detail below and a flowchart of the simulation process can be seen in Fig. 2b. The simulation started by initiating the pump and Stokes beams at the entrance of the interaction volume. The beams were then propagated a short distance Δz into the sample using diffraction theory. The resulting SRG and SRL were added to each beam as a phase contribution due to PM. The process was repeated until the end of the interaction volume at *z* = *L* was reached.

## Diffraction Theory

In general, the complex amplitude *U*(*x*,*y*,*z*) of a monochromatic light field propagating towards positive *z*-values can be written as^33^

(1) $$U\left(x,y,z\right)=\int_{-\infty }^{\infty }h\left({\text{σ}}_{\text{x}},{\text{σ}}_{\text{y}}\right)\exp[\text{i}2\text{π}\left({\text{σ}}_{\text{x}}x+{\text{σ}}_{\text{y}}y+{\text{σ}}_{\text{z}}z\right)]{\text{dσ}}_{\text{x}}{\text{dσ}}_{\text{y}}$$

where (*x*,*y*) are spatial coordinates, (σ_*x*_,σ_*y*_,σ_*z*_) are spatial frequencies, and *h*(σ_*x*_,σ_*y*_) is a function that contains the amplitudes of the wave components that make up the field.

Equation 1 satisfies the Helmholtz equation, and the spatial frequencies are restricted by ${\text{σ}}_{\text{x}}^{2}+{\text{σ}}_{\text{y}}^{2}+{\text{σ}}_{\text{z}}^{2}=1/{\text{λ}}^{2}$ , where λ is the wavelength of the light field. Assuming that the field is known at *U*(*x*,*y*,*z* = 0), then *h*(σ_*x*_,σ_*y*_) can be determined by

(2) $$h\left({\text{σ}}_{\text{x}},{\text{σ}}_{\text{y}}\right)=\int_{-\infty }^{\infty }U\left(x,y,z=0\right)\text{exp}\left[-\text{i}2\text{π}\left({\text{σ}}_{\text{x}}x+{\text{σ}}_{\text{y}}y\right)\right]\text{d}x\text{d}y$$

which is the 2D Fourier transform of *U*(*x*,*y*,*z* = 0), that is, *h*(σ_*x*_,σ_*y*_) = ℱ*{U*(*x*,*y*,*z* = 0)}. Given the field *U*(*x*,*y*,*z* = 0), the field at a position *z* =Δ*z* can thus be expressed as

(3) $$U\left(x,y,z=\Delta z\right)=\int_{-\infty }^{\infty }h\left({\text{σ}}_{\text{x}},{\text{σ}}_{\text{y}}\right)\text{exp}\left[\text{i}2\text{π}\left({\text{σ}}_{\text{x}}\text{x}+{\text{σ}}_{\text{y}}\text{y}\right)\right]\exp\left(\text{i}2{\text{πσ}}_{\text{z}}\Delta z\right){\text{dσ}}_{\text{X}}{\text{dσ}}_{\text{y}}$$

where the first exponential is recognized as the invers 2D Fourier kernel, that is, *U*(*x*,*y*,*z* = Δ*z*) = ℱ^−1^ {*h*(σ_*x*_,σ_*y*_)exp(*i*kσ_z_Δ*z*)}. This is the Fourier shifting theorem in the *z*-direction. Thus, by defining the complex wave amplitudes *U*_*P*_(*x*,*y*,*z* = 0) and *U*_*S*_(*x*,*y*,*z* = 0) for the pump and Stokes beams, respectively, the waves at *q*Δ*z*, *U*_*P*_(*x*,*y*,*z* = *q*Δ*z*), and *U*_*S*_(*x*,*y*,*z* = *q*Δ*z*) where *q* is an integer value, can be determined independently by repetitive use of Eq. 3 throughout the interaction volume.

### Phase Modulation

Because of SRS, the two fields are not independent. Therefore, after the wave has propagated a distance, Δ*z*, the resulting SRG and SRL needs to be added to the beams. The Stokes intensity in the SRS process can be expressed as^34,35^

(4) $$I_{\text{S}}=I_{\text{S}0}\exp\left(g_{\text{r}}I_{\text{P}}\Delta z\right)$$

where *I*_S0_ is the initial Stokes intensity, *g*_r_ is the Raman gain coefficient, and Δ*z* is the interaction length. The pump intensity is similarly expressed as

(5) $$I_{\text{P}}=I_{\text{P}0}\exp\left(-\frac{{\text{λ}}_{\text{P}}}{{\text{λ}}_{\text{S}}}g_{\text{r}}I_{\text{s}}\Delta z\right)$$

where *I*_P0_ is the initial pump intensity. The pump wavelength λ_P_, and the Stokes wavelength λ_S_, are included to maintain the energy balance in the interaction. If the optical wave entering the sample has a high intensity, *I*, as is usually the case in SRS, an optical Kerr effect will be induced into the sample. The Kerr effect is modeled as^36^

(6) $$n\left(I\right)=n_{1}+n_{2}I$$

where *n*(*I*) is the overall refractive index of the sample, *n*_1_ is the linear refractive index, and *n*_2_ = *n*_r_ – i*n*_i_ is the (generally complex) nonlinear refractive index. Thus, a high intensity wave propagating through a sample will cause a change in the refractive index as a function of intensity. A result of this is that the optical wave will undergo a PM which, under the first Born approximation, can be written as^36^ $U\left(\Delta z\right)=U_{\text{L}}\left(\Delta z\right)\text{exp}\left(\text{iΔ}\varphi \right)$ , where

(7) $$\Delta \varphi =k_{0}{\text{n}}_{2}I\Delta z$$

for the complex amplitude, and *k*_0_ is the wavenumber of the wave in vacuum and *U*(Δ*z*) is the linear field related to *n*_1_ in Eq. 6. Equation 7 provides a sufficient approximation as long as Δ*z* is kept sufficiently small. The nonlinear phase contribution in the Stokes and pump beams can thus be written as

(8) $$\Delta \varphi_{\text{S}}=k_{\text{S}0}n_{2}I_{\text{P}}\Delta z\approx -\text{i}k_{\text{S}0}n_{\text{i}}I_{\text{P}}\Delta z=-\text{i}g_{\text{r}}I_{\text{P}}\Delta z/2$$

and

(9) $$\Delta \varphi_{\text{P}}=-k_{\text{P}0}n_{2}I_{\text{S}}\Delta z\approx \text{i}k_{\text{P}0}n_{\text{i}}I_{\text{S}}\Delta z=\text{i}\frac{{\text{λ}}_{\text{P}0}}{2{\text{λ}}_{\text{S}0}\;}g_{\text{r}}I_{\text{S}}\Delta z$$

where *k*_S0_=2π/λ_S0_ and k_P0_ = 2π/λ_P0_ represents the wavenumbers of the waves in vacuum for the Stokes and pump beams with the wavelengths λ_S0_ and λ_P0_, respectively. Note that with these relations the absorbing part of *n*_2_ may be expressed as *n*_i_ = *g*_r_λ_S0_/4π and *n*_i_ = λ_P0_^2^*g*_r_/4π λ_S0_ for the SRG and SRL interactions, respectively. In Eqs. 8 and 9, the real part of *n*_2_ has been ignored as it is usually small and since we are mainly interested in the energy transfer between the beams. From these two equations it must be understood that the Raman gain coefficient, *g*_r_, controls the interaction between the two beams. Note that *g*_r_ is wavelength dependent so the PM only contributes when the two beams are in resonance with a molecular vibration.

### Numerical Implementation

Based on the theory in the sections above, simulations were implemented in Matlab. The numerical representation of the discretization of the sample together with images of the initial pump and Stokes beams can be seen in Fig. 2c. To simulate the beam propagation, the sample volume in Fig. 2a was split into *N* = 7500 *x*,*y*-planes along the *z*-direction, all separated by a distance Δ*z* = 50λ_S_. The total propagation length for the simulations was *L* = 174 mm. The *x*,*y*-plane used to define the initial beams was spanned by 2560 × 2160 grid points that were spaced 3.7 µm from each other in both directions. Then a 2000 x 2000 point grid was cropped from the center of the initial grid. This resulted in an *x*,*y*-plane of size 7.4 × 7.4 mm^2^. The initial beam profiles in the first *x*,*y*-plane were defined as Gaussian profiles using the equation^36^

(10) $$U_{\text{Gauss}}\left(x,y\right)=\sqrt{\frac{2P}{\text{π}}}\frac{1}{W\left(z_{\text{g}}\right)}\exp\left[-\frac{\left(x^{2}+y^{2}\right)}{W{\left(z_{\text{g}}\right)}^{2}}\right]\exp\left[-\text{i}kz_{\text{g}}-\text{i}k\frac{\left(x^{2}+y^{2}\right)}{2R\left(z_{\text{g}}\right)}+\text{i}\tan^{-1}\frac{z_{\text{g}}}{z_{0}}\right]\;$$

where *P* is the peak power of the laser pulses, $W\left(z_{\text{g}}\right)=W_{0}\sqrt{1+z_{\text{g}}^{2}/z_{0}^{2}}$ is the beam radius at position *z*_g_, $R\left(z_{\text{g}}\right)={\text{z}}_{\text{g}}\left[1+z_{0}^{2}/z_{\text{g}}^{2}\right]$ is the wavefront radius of curvature, $W_{0}=\sqrt{\text{λ}z_{0}/\text{ π }}$ is the waist radius of the beam, and *z*_0_ is half the focus depth; *z*_g_ is a coordinate defined for the Gaussian beam so that the beam waist is located at *z*_g_ = 0. The Stokes beam was made to propagate collimated through the sample by setting the beam radius to *W*_S0_ = 4.55 mm. The pump beam was focused to a beam with a waist radius of *W*_P0_ = 11 μm, located in the middle of the sample at *z* ≈ 85 mm. The parameters regarding the laser beams wavelengths, pulse energies and the refractive indices in ethanol were selected to correspond with the experimental values. Values of *n*_2_ for ethanol have been reported^37–39^ and were used as a guide the initial value of *n*_i_, which was then tuned to fit the experiments. In Table I, the final beam parameters are summarized.

**Table I.** The final beam parameter values used in the computer simulations, *n*_*i*_ is the imaginary part of the nonlinear refractive index *n*_2_, that is, *n*_2_ = *n*_*r*_ – i*n*_*i*_.

Parameter	Pump beam	Stokes beam
Pulse energy	1.1, 6.5, 16.5, 23.5, 27.0 mJ	0.36 mJ
Wavelength	532 nm	630.45 nm
Refractive index^40^	1.3637	1.3605
*n*_*i*_	3.6 × 10^−21^	3.6 × 10^−21^
Beam waist radius	11 μm	4.55 mm (collimated)

## Results and Discussion

**Figure 3.**
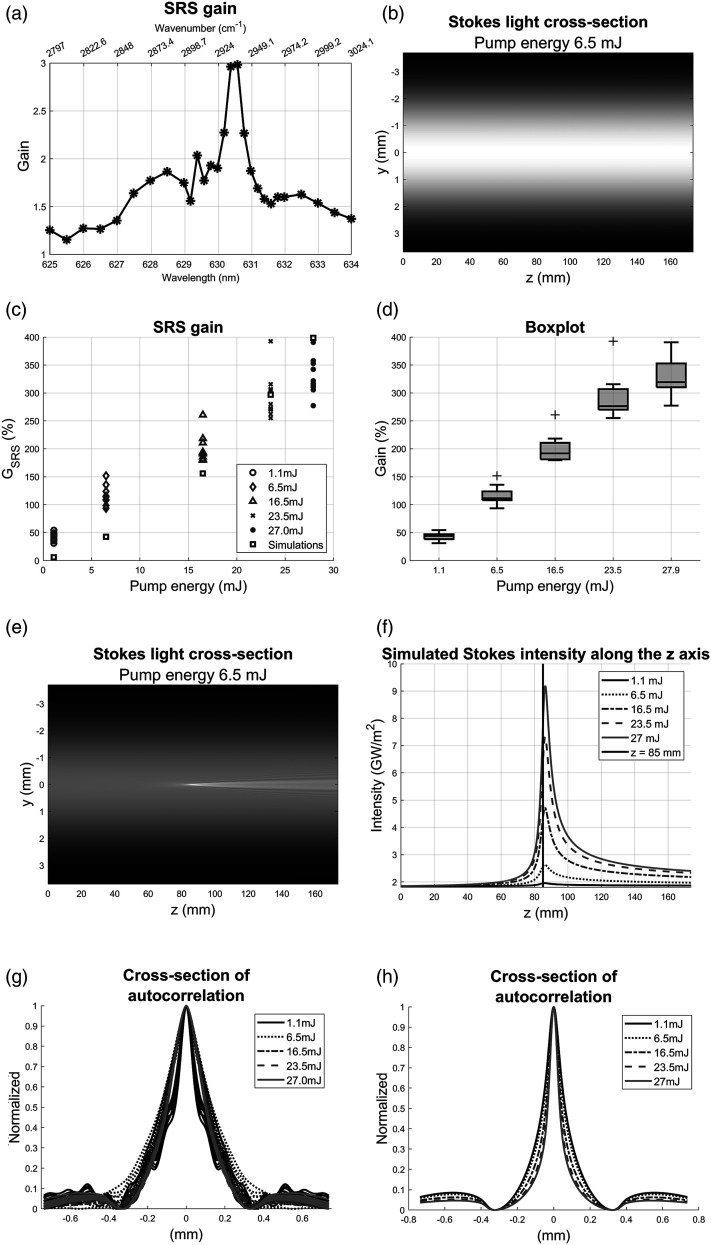
(a) The experimental SRS gain, *G*_SRS_, as a function of Stokes wavelength. (b) The simulated SRS gain, *G*_SRS_, in the *y*,*z*-plane when *n*_i_ = 0 and *E*_pump_ = 6.5 mJ. (c) The SRS gain; the simulation data is marked as black squares; the gray markers show the experimental data for different pump energies. (d) A box plot of the experimental *G*_SRS_ in (c), plus signs indicate outliers. (e) The simulated Stokes intensity in the *y*,*z*-plane. (f) The simulated Stokes intensity along the *z*-axis. The vertical line marks the position of the pump beam focus. (g) The cross-section of the autocorrelation from the experiments. (h) The cross-section of the autocorrelation from the simulations.

To ensure that the signal measured was an amplification of the SRS process and not caused by thermal lensing, a series of measurements for different Stokes wavelengths was performed. In Fig. 3a, the measured SRS gain is shown as a response to a wavelength sweep of the Stokes beam across the theoretical resonance wavelength of 630.45 nm. It is seen that if the wavelength of the Stokes beam did not correspond to the selected Raman shift the SRS signal was lost. It may therefore be concluded that the signal in the images when the wavelength of the Stokes beam was 630.45 nm came from the SRS process.

Two experiments were performed. In the first experiment, the sample setup in Fig. 1a (panel 5A) was used. The pump energy *E*_pump_ was varied to investigate the change in G_SRS_ and the width of the SRS peak. The pump pulse energies used were *E*_pump_ = 1.1, 6.5, 16.5, 23.5, and 27.0 mJ and 10 measurements were made for each pump energy. First, a simulation for the pump energy *E*_pump_ = 6.5 mJ and *n*_i_ = 0 was performed, the resulting simulated Stokes intensity in the *y*,*z*-plane can be seen in Fig. 3b. The beam propagates from the left to the right. It can be seen that the beam has an even Gaussian profile through the whole sample. This shows that no SRS is generated in the simulation when the interaction between the beams and the sample is removed. Computer simulations for each pump energy were then performed with *n*_i_ = 3.6 × 10^−21^ and the SRS gain, *G*_SRS_, from experiments and simulations can be seen in Fig. 3c. The experimental SRG are marked as circles, dimonds, triangles, crosses and dots. Meanwhile the simulated SRG are marked as black squares. For *E*_pump_ = 1.1, 6.5, and 16.5 mJ, the simulated SRG are lower compared to the experimental SRG. For *E*_pump_ = 23.5, 27.0 mJ, the simulated SRG lies in the same region as the experimental SRG. The simulated curve follows an exponential trend meanwhile the experimental curve is linear. The exponential behavior of the simulations is a result of the use of exponential functions in the theory of the gain. The linear trend in the experimental data is likely to be caused by a saturation effect due to the high intensity beams, which has not been taken into consideration in the simulations. The spread in the experimental data seems to increase with increasing pump energy so a box plot was used to visualize the spread, see Fig. 3d. Three outliers, for *E*_pump_ = 16.5, 23.5, and 27.0 mJ, can be seen as red plus signs. The whiskers and first and third quantile indicate an increase in the data spread as the pump energy increases. The data spread of the experimental data is most likely caused by the pulse-to-pulse energy fluctuations of the Stokes beam.

The interaction between the pump (*E*_pump_ = 6.5 mJ) and the Stokes beam in the z,y-plane is shown in Fig. 3e. As the beam comes closer to the center the SRG can be seen as a brighter white spot in the middle. The light diverges as the beams continue to propagate through the sample, which is a result of diffraction. The image shows that most of the SRS process takes place close to the middle of the sample, where the pump beam focus is located. To compare the SRG between the five pump energies, the intensity along the *z*-axis was extracted from the simulated data and the result is shown in Fig. 3f. The black line at *z* = 85 mm marks the position of the pump beam focus. At the entrance of the sample, *z* = 0, the Stokes intensities were the same for the five different pump energies. Around *z* = 40 mm, the intensity starts to increase for *E*_pump_ = 16.5, 23.5, and 27.0 mJ. For *E*_pump_ = 6.5 mJ, the SRG becomes visible at *z* =70 mm. For all pump energies, except the lowest one, the intensity increased sharply just before the pump beam focus and came to a peak after the pump beam focus and to quickly decreased again. However, it should be noted that the growth of the Stokes light is a cumulative effect; the intensity at one point is a result of the generated light at that point and the previously generated light. After the pump beam focus the light generation almost stops and the intensity drops as the beam diverges with continued propagation. A small peak in intensity can also be seen for the lowest energy. The graph indicates that most of the SRG was generated close to the pump beam focus for all pump energies. Figure 3f also shows that the position of the Stokes maximum lies deeper into the sample compared to the position of the pump beam focus. The difference in peak position relative to the position of the beam waist of the pump beam for *E*_pump_ = 1.1 mJ was 0.88 mm and for *E*_pump_ = 27.0 mJ was 1.31 mm. The width of the SRS peak was investigated using autocorrelation. From an SRS image, looking like the one displaced in the left image in Fig. 1c, a 200 × 200-pixel window containing the SRG signal was cropped and a correlation image was calculated. From the resulting 399 × 399 pixel image, the center row was extracted and normalized. The SRS image from the simulation was calculated by extracting the Stokes intensity at the *x*,*y*-plane where *z* = 85 mm (the plane where the pump beam focus was positioned) and then dividing it by the initial Stokes beam. The cross-section of the autocorrelations of the experimental data and simulated data can be seen in Figs. 3g and 3h, respectively. In Fig. 3g each line style represents a pump energy, and every line is a separate measurement. The curves are similar to each other, but the 1.1 mJ curve shows oscillations that are caused by a stripe pattern in the SRS images. The mean full width at half-maximum (FWHM) of the peaks is 0.21 mm. The curves in Fig. 3h for the simulations are all very similar to each other suggesting that the width of the SRS peak does not increase with increasing pump energy. The mean FWHM of the peaks are 0.09 mm. The filtering of the experimental data widens the SRS peak which causes a larger FWHM value of the autocorrelation. The Gaussian filter kernel used had a window size of 17 pixels, with a standard deviation of four pixels, which lead to a widening around 8–12 pixels of the SRS signal. With one pixel in the SRS images corresponding to 3.7 μm, the FWHM is about 30–44 μm broader after the filtering. Subtracting this from 0.21 mm brings the value down to 0.17–0.18 mm, which is closer to the FWHM from the simulation. It is also probable that the imaging of the SRS signal to the camera causes some broadening of the peak. Note that the FWHM of the autocorrelation is twice as wide as the physical width of the spatial distribution. If the FWHM for the simulation is divided by two the width becomes 0.045 mm, or 45 μm. The radius is then 23 μm which is 12 μm larger than the beam waist radius used in the simulation, 11 μm. This is probably the result of that SRS starts to generate just before the pump beam reaches its focal point. Calculating the physical width for the experiment yields a width of 40–45 μm, which is 17–22 μm larger compared to the simulated width of 23 μm. The difference suggests that the pump beam waist radius in the simulations is somewhat too small and consequently the peak intensity becomes higher in the simulations as compared to the experiments.

**Figure 4.**
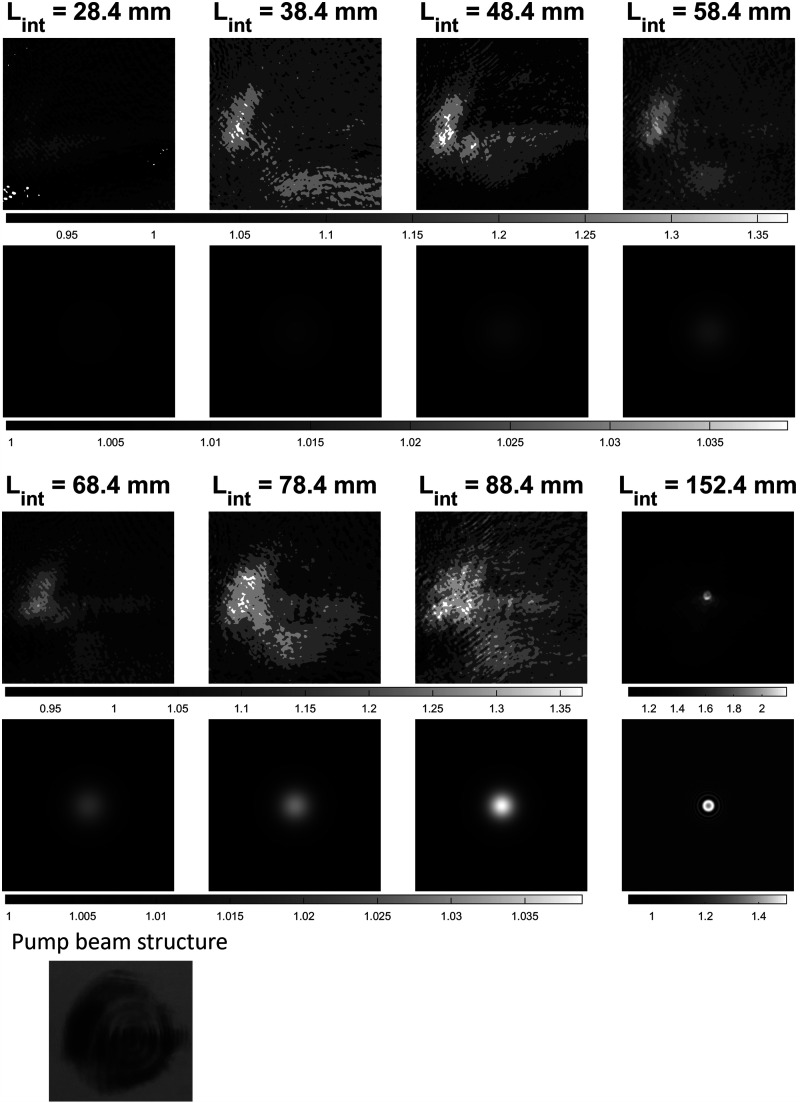
The experimental SRS images above the corresponding simulated images for the interaction length *L*_*Int*_ = 28.4, 38.4, 48.4, 58.4, 68.4, 78.4, 88.4, and 152.4 mm, respectively. The color bar for the experimental images is the same, except for *L*_*Int*_ = 152.4 mm, which has a different scale. The same is true for the simulated images. A photograph of the burn pattern of the laser beam before lens (2) in Fig. 1a, the radius was around 3.5–4 mm.

For the second experiment, the sample setup displayed in Fig. 1a (panel 5B) was used. The pulse energy for the pump was set to 16.5 mJ and the mirror submerged in the ethanol was moved to *L*_Int_ = 28.4, 38.4, 48.4, 58.4, 68.4, 78.4, and 152.4 mm, respectively. Note that for the last interaction length, the mirror was placed behind the pump beam focus. Ten measurements were performed for each interaction length and averaged into one SRS image. The physical sample setup was mimicked in computer simulations and the results can be seen in Fig. 4. A photograph of the burn pattern on photo sensitive paper of the pump beam profile before lens (2) is also provided. The radius of the beam was about 3.5–4 mm. For each interaction length the experimental SRS image is displayed as a contoured plot above the corresponding simulated image. Note that for *L*_Int_ = 28.4–88.4 mm the experimental images all have the same scaling meanwhile the simulated SRS images have the same scaling. The colorbar can be seen below the images. Note that for *L*_Int_ = 152.4 mm the images have a separate colorbar. Starting with the experimental results, the SRG can be seen as the brighter areas in the images. In the experimental images for *L*_Int_ = 28.4–88.4 mm, a slightly brighter area centered at the left edge of the images can be seen. Comparing this to the pump beam pattern, these areas seem to correspond to the high intensity area of the burn pattern seen in Fig. 4. This suggests that small amounts of SRS are generated early in the sample in the areas of the pump beam with the highest intensity. The SRS image for *L*_Int_ = 152.4 mm looks very different as the SRG now has the shape of a small dot. However, note that the shape of the lighter areas surrounding the dot resembles that of the pump beam pattern due to the large difference in the signal amplitude, between 1.05 and 1.35 compared to a value of two. In the results from the simulation, it can be seen that small amounts of SRS were generated early in the sample. As the interaction length increased the center of the images became brighter. Comparing the experimental results with the simulated ones, the area of increased intensity at the regions of higher pump beam intensities are similar. The difference in shape is due to the uneven pump beam profile. The simulated image for *L*_Int_ = 152.4 mm has a striking resemblance to the experimental one showing a sharp point.

## Conclusion

In this work, the SRS generation of the 2934 cm^−1^ Raman line of ethanol was investigated. In the simulations, the beam propagation was modeled using diffraction theory and the nonlinear interaction between the laser beams and the sample was modeled as a phase modulation due to the induced Kerr effect in the medium. Experiments were carried out by illuminating a cylindrical volume of the ethanol sample with an expanded and collimated Stokes beam while the pump beam was focused into this region at different depths. The change in spatial distribution of the SRS gain due to an increase in pump power was investigated. The spatial propagation of the generated SRS signal was investigated by submerging a dichroic mirror into the sample to interrupt the process at specified interaction lengths. Comparisons between experiments and simulations of the generated SRS at different interaction lengths showed that most of the SRS gain is generated close to the focal point of the pump laser. The width of the SRS gain did not change significantly with increasing pump energy and the mean FWHM of the experiments and simulations were 0.21 mm and 0.09 mm, respectively. The difference is mainly caused by the filtering of the experimental data and the imaging setup, but part of the difference may be explained by the difference in the original beam profile. The simulations also showed that the position of the maximum intensity in the Stokes gain comes after the maximum intensity of the pump beam, which confirms the theoretical prediction made by Carman et al.^19^

Knowledge on the position is paramount for the spatial determination of the SRG for accurate imaging. This knowledge will help in the fusing of SRS with imaging for direct 3D imaging of species distrubutions.

The experimental setup presented in this paper is most suitable for measurements on highly Raman scattering samples such as pure liquids and gases due to the high pulse energies and long pulse durations of the laser. For more fragile samples a shorter pulse duration is recommended. The use of an expanded Stokes beam and focused pump beam makes it easy to ensure that the two beams overlap each other.
